# Return to an extreme environment exposure after pulmonary infection: SCUBA diving as a model?

**DOI:** 10.14814/phy2.70862

**Published:** 2026-04-07

**Authors:** Danilo Cialoni, Andrea Brizzolari, Simona Mrakic‐Sposta, Alessandra Vezzoli, Cinzia Dellanoce, Massimo Pieri, Riccardo Pelliccia, Chiara Petrassi, Gerardo Bosco, Alessandra Barassi, Alessandro Marroni

**Affiliations:** ^1^ Department of Human Sciences and Promotion of the Quality of Life San Raffaele University of Rome Roma Italy; ^2^ Department of Health Sciences Università Degli Studi of Milan Milan Italy; ^3^ Institute of Clinical Physiology National Research Council (IFC‐CNR) Milan Italy; ^4^ DAN Europe Research Division Roseto degli Abruzzi Italy; ^5^ Department of Medicine and Aging Sciences University G. d'Annunzio of Chieti‐Pescara Chieti Italy

**Keywords:** diving, glycocalyx, inflammation, lung, nitric oxide, oxidative stress

## Abstract

The human body implements several adaptive mechanisms to perform in extreme environments. Increasingly, individuals return to such conditions after pulmonary infections, raising concerns about potential long‐term respiratory consequences. This study aims to investigate the return to SCUBA diving after pulmonary infection, using COVID‐19 as a model to provide evidence for safe reintegration into extreme environments, with a focus on vascular, oxidative and vascular stress. Twenty‐three expert SCUBA divers who had symptomatic COVID‐19 (SARS‐CoV‐2 Alpha variant) were enrolled as studied group (COV‐19). None required intensive care, but all showed signs of lung inflammation. A control group (CTR) included nine male divers. They performed three dives at increasing depths (−10, −20, and −40 m). Lung function, nitric oxide derivatives (NOx), exhaled FeNO, oxidative stress biomarkers such as reactive oxygen species (ROS), 8‐isoprostante, total antioxidant capacity (TAC) and TAC main components (albumin and uric acid), inflammatory response indicators including interleukin‐6 (IL‐6), IL‐1beta, and tumor necrosis factor‐alpha (TNF‐alpha) and glycocalyx indexes were measured before and after each dive. No differences in FeNO, NOx, or lung function were observed between groups. However, COV‐19 divers showed higher ROS, 8‐isoprostane, IL‐6, IL‐1β, and TNF‐α than the CTR divers, suggesting persistent inflammatory alterations. TAC remained unchanged. Significant differences emerged in glycocalyx markers. These results suggest an overall return to normal adaptation to diving after pulmonary infection. However, persistent inflammatory differences highlight the need for caution and personalized evaluation based on disease severity and recovery.

## Graphical abstract

Graphical overview of the experimental protocol used to investigate post‐COVID physiological responses during scuba diving at three different depths.

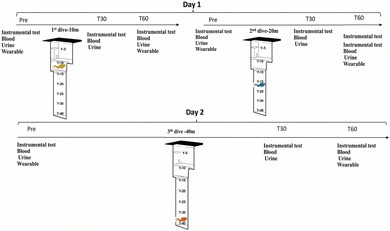

## 1 INTRODUCTION

Sports in extreme environments and/or conditions represent an array of physical activities that differ from traditional sports, continuing to attract a strong following. These sports, such as snowboarding, freeskiing, climbing, base jumping, kayaking, SCUBA diving, involve a variety of unpredictable, often inhospitable environments, variable external conditions, high speed, stunts, and specialized equipment (Gomez & Rao, 2016). In order to counteract environmental‐related stressors, the human body relies on a highly coordinated set of adaptive mechanisms, whose efficiency is particularly critical within the cardio‐respiratory system (Pendergast & Lundgren, 2009). Exposure to immersion immediately induces a cephalad redistribution of blood volume, with displacement from the dependent limbs toward the thoracic cavity and a consequent rise in central venous pressure and intrathoracic blood volume (Arborelius et al., 1972). This enhanced venous return increases cardiac preload, thereby augmenting stroke volume and overall cardiac output, while perfusion may be redistributed to the respiratory and myocardial musculature in order to sustain the effort associated with intensified ventilatory and circulatory work (Hajduczok et al., 1987). Furthermore, hydrostatic compression of the thoracic cage, imposed by the surrounding environmental pressure, generates an additional elastic load on the chest wall and promotes a pattern of negative‐pressure breathing (Song et al., 1969). The resultant static lung loading imposes significant mechanical stress on the respiratory system, leading to measurable alterations in end‐expiratory lung volume and modifying pulmonary mechanics in a way that may exacerbate strain on both alveolar and airway structures. In this physiological context, the fractional concentration of exhaled nitric oxide (FeNO) has been proposed as a sensitive, non‐invasive biomarker capable of detecting and monitoring the persistence of alveolar and bronchiolar inflammatory processes (Cameli et al., 2021). NO plays a key role in the pulmonary adaptation of subjects exposed to high hydrostatic pressure (Theunissen et al., 2013): a remarkable increase of NO derivatives, nitrates + nitrites (NOx), during the deep phase of SCUBA (Cialoni et al., 2019) and Breath‐Hold (BH)‐dives (Cialoni et al., 2020) was found. Oxidative stress, widely investigated in underwater activities (Brubakk et al., 2005; Obad et al., 2010), both in SCUBA (Cialoni et al., 2019; Theunissen et al., 2013) and BH‐diving (Cialoni et al., 2020; Mrakic‐Sposta et al., 2019; Solich‐Talanda et al., 2021), can suppress endothelial nitric oxide synthase (eNOS) production leading to NO deficiency (Tousoulis et al., 2010). Diving‐related hyperoxia (Kahle & Cooper, 2022; Wingelaar et al., 2017) can induce oxidative stress (Perovic et al., 2014), caused by the increase of reactive oxygen species (ROS) production (Obad et al., 2010; Perovic et al., 2014; Radojevic‐Popovic et al., 2015; Radojevic‐Popovic et al., 2016; Salah et al., 2023) and in order to control oxidative stress, endogenous antioxidant systems are activated (Cialoni et al., 2019; Ferrer et al., 2007; Mrakic‐Sposta et al., 2024).

BH‐diving exacerbates these mechanisms because the lack of pulmonary pressure compensation, together with repeated cycles of hypoxia/reoxygenation, promotes ROS production and activates pro‐inflammatory mediators (Elia et al., 2021; Mrakic‐Sposta et al., 2019). The diving reflex amplifies these effects via intense peripheral vasoconstriction and increased sympathetic tone, redistributing blood toward vital organs and concentrating oxidative and metabolic stress locally (Cialoni et al., 2020, 2021; Elia et al., 2021). Therefore, BH‐diving provides a relevant experimental model to study mechanisms of pulmonary and vascular injury and repair under intermittent hypoxia (Vezzoli et al., 2024).

In several cases practitioners exposed themselves to extreme conditions after healing from pulmonary infections of various kinds and in the last years the Corona Virus Disease (COVID‐19) pandemic, increased the number of people exposed to this risk. Altered pulmonary functions induce mitochondrial dysfunction, NADPH‐oxidase activation and oxidative stress, and therefore initiates a feedback loop promoting a chronic state of inflammation and/or endothelial dysfunction, inducing inflammatory cytokines production including interleukin‐6 (Il‐6), IL‐1β and tumor necrosis factor‐α (TNF‐α) (Chang et al., 2020; Del Valle et al., 2020; Tripathy et al., 2021). During sepsis, the glycocalyx, a gel‐like layer lining the luminal surface of endothelial cells (Weinbaum et al., 2007), is degraded via inflammatory mechanisms activated by ROS, IL‐1β and TNFα, leading to vascular hyper‐permeability, unregulated vasodilation, microvessel thrombosis, and augmented leukocyte adhesion (Uchimido et al., 2019). These conditions lead to impaired alveolar oxygenation, hypoxemia, and acidosis causing low peripheral oxygen saturation (SpO_2_) (Dominguez‐Nicolas & Manjarrez, 2021) and promoting ROS production (Trentadue et al., 2012). ROS exacerbate the expression of IL‐1, IL‐6, and TNFα, and of inducible NOS (iNOS) via activation of the NF‐κB pathway (Nanduri et al., 2008; Takada et al., 2003), generating more free radicals (Nanduri et al., 2008). Neopterin can increase during systemic oxidative stress (Baxter‐Parker et al., 2019; Lindsay & Gieseg, 2020; Mrakic‐Sposta et al., 2019) as an indication of an increase in inflammatory condition (Murr et al., 2002).

All the above confirms that the main organs and phenomena affected in COVID‐19 are the same involved in the physiological response to diving activities and suggests that diving could be an interesting physiological and pathophysiological study model to investigate the return to extreme environment activities after pulmonary infections.

Pulmonary infections, whether of viral or bacterial origin, represent a recurrent challenge for individuals intending to resume demanding physical activities. Despite differences in etiology, COVID‐19 and common pulmonary infections share several clinical and functional features that justify their comparison (Di Mitri et al., 2021; Ibanez‐Prada et al., 2023; Tian et al., 2020). Both conditions involve an acute inflammatory process in the lungs, which may impair alveolar gas exchange and lead to transient or prolonged hypoxaemia. Systemic manifestations such as fever, fatigue, and malaise are also characteristic of both entities. In addition, pulmonary sequelae, including persistent airway inflammation and reduced diffusion capacity, have been reported after recovery from either COVID‐19 or conventional pneumonia (Patton et al., 2024; Ribeiro Carvalho et al., 2024). While some patients regain normal respiratory function within weeks, others exhibit residual impairment for months. This variability complicates the determination of safe thresholds for return to strenuous activities, particularly those involving exposure to extreme environments (Elia & Gennser, 2020). SCUBA diving offers a pertinent model in this regard, as it places unique stress on the respiratory system through increased ambient pressure, immersion physiology, and rapid changes in gas dynamics. Any residual pulmonary vulnerability may increase the risk of barotrauma, hypoxia, or decompression illness. COVID‐19 has been employed as a model pathology to study this issue, not only because of its global prevalence but also due to the extensive data generated on post‐infection sequelae (Morin et al., 2022).

The primary objective of our study is to identify potential biomarkers and surrogate endpoints capable of revealing persistent structural and functional abnormalities, both systemic and lung‐specific, beyond the phase of apparent “clinical recovery.” Such residual alterations may include ongoing low‐grade inflammation, impaired alveolar‐capillary diffusion, or subtle ventilatory limitations, which are not always detectable through routine clinical assessment. Persisting abnormalities acquire particular relevance in the context of hyperbaric exposure. Indeed, immersion and SCUBA diving impose unique physiological stressors, including elevated ambient pressure, altered gas dynamics, and immersion‐induced cardiovascular shifts. Rapid pressure variations during ascent and descent can exacerbate otherwise subclinical pulmonary vulnerabilities, thereby increasing the likelihood of barotrauma, pulmonary oedema, or decompression‐related complications. Hence, the identification of reliable biomarkers may inform evidence‐based clearance protocols, refine individualized risk assessment, and ultimately contribute to safer return‐to‐dive strategies after pulmonary infection.

## 2 MATERIALS AND METHODS

### 2.1 Subjects

A total of 23 expert SCUBA divers (19 males and 4 females), who suffered from pulmonary symptomatic infection of COVID‐19 SARS‐CoV‐2 Alpha variant, were included in the studied group (COV‐19) and evaluated during three dives. None of the COV‐19 subjects required intensive care, but all needed treatment in the emergency room or consultation with an emergency physician due to varying degrees of lung symptoms that included coughing, difficulty breathing, and reduced blood oxygen saturation. The control group (CTR), consisting of 9 male divers who had not contracted COVID‐19, was also part of the study. Neither the COV‐19 nor the CTR participants were vaccinated at the time of the study which was conducted between April 2021 and November 2021. All COV‐19 subjects are included in the study 3 months after the resolution of symptoms and not over 4 months. No subject reported previous episodes of decompression illness (DCI), historical or clinical evidence of arterial hypertension, cardiac, pulmonary or any other significant disease. None of them took any drug, suffered any acute disease during the 15 days before the experiment, or was exposed to high altitude in the 7 days before the experiment. Subjects were allowed to avoid foods naturally rich in nitrates (e.g., red meat and leafy green vegetables) during the 24 h preceding the experiment. All participants declared that they did not use any dietary supplements and followed their usual diet. No additional dietary standardization was applied.

The day before the experiment, every volunteer did a nasopharyngeal swab to exclude any new infections of COVID‐19 and was allowed to drink coffee, smoke, or drink alcohol before. All the divers received an explanation about the study's purposes, risks and benefits, were familiarized with the experimental protocol, and read and signed a specific informed consent form before the experiment. Subjects were asked to avoid moderate/intense exercise in the 48 h before the experiment. The study was conducted in accordance with the Helsinki Declaration and was approved by the Ethical Committee of Università degli Studi di Milano, Italy (Aut. n° 37/17) (World Medical Association, 2013).

### 2.2 Dive protocol

The divers did three dives, each with a total dive time of 40 min breathing air as gas mixture. All dives were performed in the swimming pool “Y‐40 The Deep Joy” (Montegrotto Terme, Italy), filled with thermal water maintained at a temperature of approximately 32°C–33°C and with a maximum depth of 42 m, ensuring stable environmental conditions throughout the experimental sessions.

Diving profiles were the following:

Dive 1 (Day 1): a dive at 10 msw for a bottom time (time at maximum depth, BT) of 35 min with a safety stop of 5 min at 5 msw.

Dive 2 (Day 1, 5 h after the first dive): a dive at 20 msw for a BT of 35 min with a safety stop of 5 min at 5 msw.

Dive 3 (Day 2): a dive at the depth of 40 msw with a bottom time of 5 min, and the safety stop of 5 min at 5 msw.

No extra time decompression was included in the planning of the protocol and there is no need for it.

Diving parameters, including depth, diving time and inert gas supersaturation Gradient Factor (GF) were recorded for each dive using a diving computer set on the Buhlmann ZHL16‐C algorithm. All dive profiles were downloaded to calculate the Maximum GF to estimate inert gas supersaturation and to compare diving exposure in the subjects (Cialoni et al., 2017).

**FIGURE 1:**
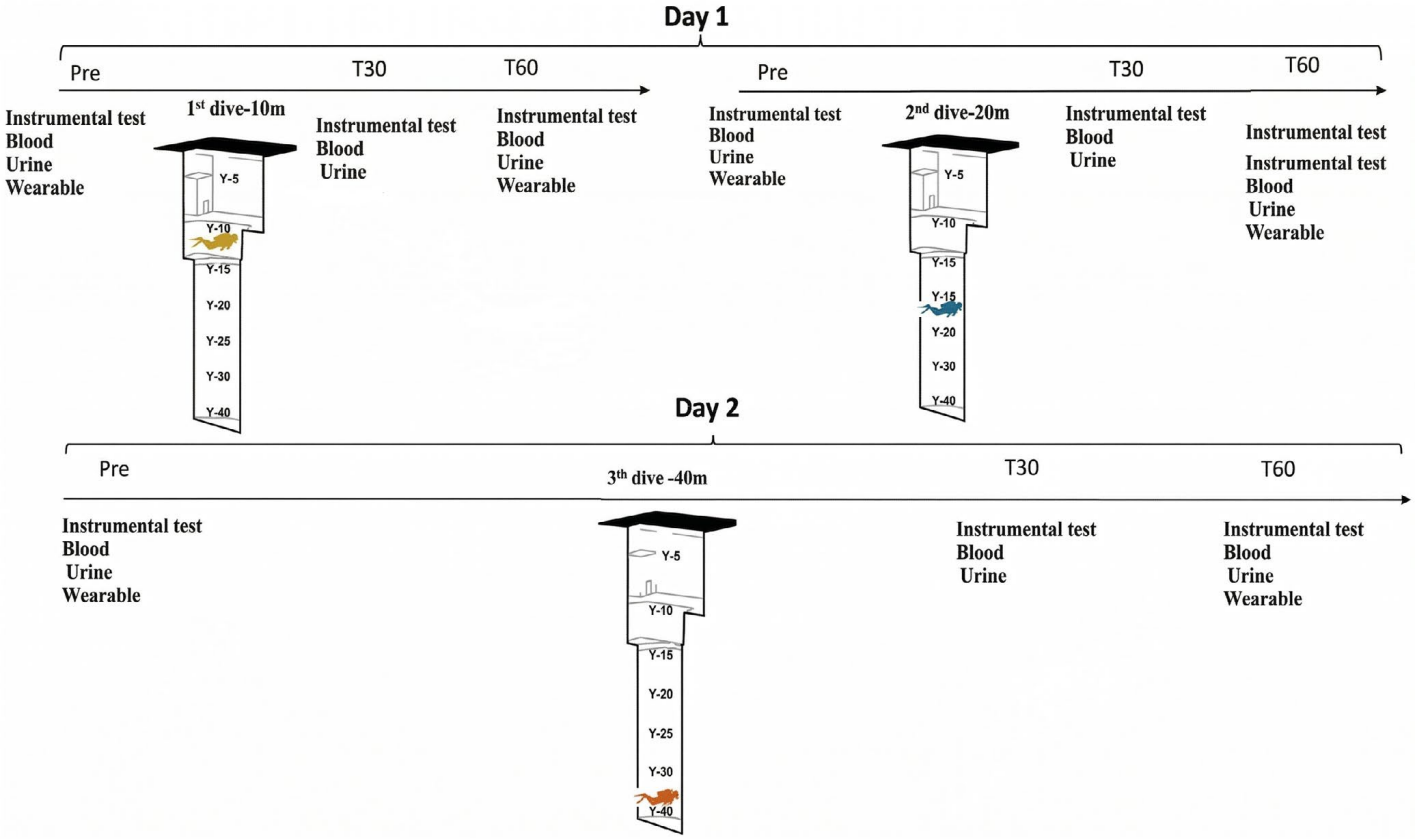
Experimental protocol.

Figure 1 reported the experimental protocol. All the different parameters were investigated according to the following timing:

- Pre‐exposure: 30 min before the dive;
- T30: 30 min after surfacing;
- T60: 60 min after surfacing.

### 2.3 Sample collection

We collected blood samples in three different tubes (Vacutainer, Becton, Dickinson and Company, Franklin Lakes, NJ, United States), containing EDTA, Lithium Heparin for plasma, and Citrate gel for serum preparation. Plasma/serum was separated from the cell component by centrifugation (3000 rpm for 10 min) (Firuzi et al., 2006).

Also, urine samples were collected via voluntary voiding into a sterile container provided to the subjects and were used to determine lipid peroxidation (8iso‐pGF2α), neopterin, and creatinine concentrations. Multiple aliquots of plasma and urine were immediately frozen and stored at −80°C.

### 2.4 NO derivatives

Exhaled FeNO was measured before and after every dive using a FeNOBreath (Bedfont Scientific, Maidstone, UK). Divers expired into a buccal connected to the instrument for 10s. Results are expressed as FeNO ppb.

Plasmatic nitrate and nitrite (NOx) were measured after deproteinization with acetonitrile to precipitate proteins. Standard solutions of NaNO_3_ were used to build the nitrate (NO_3_) calibration curve. NOx were determined according to Brizzolari et al. (Brizzolari et al., 2021). The inter‐assay coefficient of variation (CV) was ≤6%.

### 2.5 Oxidative stress markers

Electron Paramagnetic Resonance Spectroscopy X‐band (EPR, 9.3 GHz) (E‐Scan Bruker, Billerica, MA, USA) was used to assess ROS production at 37°C by Temperature and Gas Controller “Bio III” (Noxigen Science Transfer & Diagnostics GmbH, Elzach, Germany), interfaced with the E‐Scan. ROS production assessment methods were previously described (Brizzolari et al., 2023; Mrakic‐Sposta et al., 2012; Mrakic‐Sposta et al., 2019). Briefly, CMH (1‐hydroxy‐3‐methoxycarbonyl‐2,2,5,5‐tetramethylpyrrolidine) spin trap probe was used for ROS determination and stable radical CP• (3‐Carboxy2,2,5,5‐tetramethyl‐1‐pyrrolidinyloxy) as an external reference to convert ROS determinations into absolute quantitative values (μmol min^−1^). The best fitting straight lines (*R* ^2^ = 0.99) were found almost superimposable: about the 0.5% discrepancy in the ROS absolute production rate (μmol min^−1^) was calculated between the measurements.

Serum TAC was investigated using Trolox Equivalent Antioxidant Capacity (TEAC) assay (Re et al., 1999), according to Cialoni et al. (Cialoni et al., 2020). Standard solutions of Trolox (238813‐1G, Sigma‐Aldrich [Merk Group] Darmstadt, Germany) were prepared to build the calibration curve. The intra‐assay CV was ≤7%.

Albumin and uric acid were measured in serum using VITROS® 5600 (Ortho‐Clinical Diagnostics, High Wycombe, United Kingdom). Briefly, 500 μL of each sample was placed in a plastic test tube. The test tubes were allocated in the auto sampler. The intra assay CV was <1.8%.

Lipid peroxidation was assessed in urine by a competitive immunoassay kit (Cayman Chemical item No. 16350, Ann Arbor, Michigan, USA) for determination. Samples were read at a wavelength of 512 nm, as previously described, and the inter‐assay CV was in the range indicated by the manufacturer (Mrakic‐Sposta et al., 2019; Vezzoli et al., 2016).

### 2.6 Inflammatory markers

Plasmatic interleukins IL‐6 (Cayman Chemical item No. 501030, Ann Arbor, Michigan., USA), TNF‐α (Fine Test item No. EH0302, Wuhan China), IL‐1β (Cayman Chemical item No. 589201, Ann Arbor, MI, USA) were measured using human interleukins ELISA kits, according to the manufacturer's instructions. The determinations were assessed in duplicate, and the inter‐assay CV was in the range indicated by the manufacturer (Mrakic‐Sposta et al., 2015; Mrakic‐Sposta et al., 2020).

### 2.7 Glycocalyx markers

Heparan sulfate and syndecan‐1 (Fine Test, Wuhan China, item No. EH0278 and No. EH4010, respectively) plasmatic levels were measured using human ELISA kits according to the manufacturer's instructions. The determinations were assessed in duplicate, and the inter‐assay CV was in the range indicated by the manufacturer.

### 2.8 (renal function)

Creatinine and neopterin urinary concentrations were measured by high‐performance liquid chromatography (HPLC) method, as previously described (Brizzolari et al., 2023; Mrakic‐Sposta et al., 2020). The calibration curve was linear over the range of 0.125–1 μmol/L. Inter‐assay and intra‐assay CV were <5%.

### 2.9 Lung measurements

Spirometry was measured with a full inhalation, followed by a forced expiration that rapidly empties the lungs. Expiration is continued for as long as possible; these efforts are recorded and graphed. Calibration was performed using a 3‐L syringe to deliver three relatively constant flows at a low flow, then three at a mid‐range flow and finally three at a high flow. Calibration was done the day before the test by the dealer.

Lung function tests were performed by a trained expert physician using a Spirobank II spirometer (MIR, Berlin Wisconsin, USA) studying the following parameters: Vital Capacity (VC), the total amount of air exhaled after maximal inhalation, Forced Vital Capacity (FVC), Forced Expiratory Volume in 1 s (FEV_1_), FEV_1_/FVC ratio, and Forced Expiratory Flow between 25% and 75% of vital capacity (FEF_25‐75_).

Ultrasound lung comets (ULCs) were detected by chest echography according to Frassi et al. (Frassi et al., 2008).

### 2.10 Wearable sensor parameters

A sensorized T‐shirt able to record ECG, hearth breath rate (BR), and posture (Howdy senior, medical device class IIB manufacturer Comftech srl, Monza, Italy) was worn by the divers before and after the dive (T30). The acquisition system consists of an electronic board equipped with a microcontroller and a Bluetooth Low Energy (BLE) transceiver.

### 2.11 Other parameters

Echocardiography images were recorded using a commercially available instrument (GE Versana Active 2020 4D ready Echograph) with a cardiac probe 3Sc‐RS (2.5–3.5 MHz) to detect circulating bubbles according to the Eftedal‐Brubakk scale (Eftedal & Brubakk, 1993).

### 2.12 Statistical analysis

Data are presented as mean ± standard deviation (SD) for parametric data and median or range for non‐parametric data. The D'Agostino and Pearson normality test was used to assume a Gaussian distribution. Then, data were analyzed by either the one‐way ANOVA or the Friedman test for multiple comparison of parametric and non‐parametric data respectively, and Bonferroni post‐hoc test and Bonferroni post‐hoc was performed when appropriate. A probability lower than 5% was assumed as the threshold to reject the null hypothesis (*p* < 0.05). Data were analyzed using the GraphPad Prism software version 10.1.1 (GraphPad Software Inc., San Diego, CA, USA).

## 3 RESULTS

All the volunteers completed the experiment without DCI, pulmonary and/or ear barotraumas episodes or other health trouble. Anthropometric data and the diving profile of all the dives are shown in Table 1 both for COV‐19 group and CTR group.

**TABLE 1:** Comparison of anthropometric data and diving profile between the two investigated groups.

	COV‐19 (19 M/4F)	CTR (9 M/0F)	*p* value
Anthropometric parameters			
Age (years)	51.8 ± 10.5	43.6 ± 14.2	n.s
Height (cm)	178.2 ± 8.2	182.2 ± 5.3	n.s
Weight (kg)	83.2 ± 13.8	87.8 ± 12.6	n.s
BMI (kg/m^2^)	26.2 ± 3.9	26.4 ± 3.8	n.s
Heart Rate (bpm)	91.2 ± 16.0	89.4 ± 5.6	n.s
*Diving parameters*			
Dive 1			
Maximum depth (msw)	11.7 ± 1.0	10.9 ± 0.3	n.s
Diving time (min)	44.0 ± 7.0	40.55 ± 2.8	n.s
Temperature (°C)	32.4 ± 7.2	33.7 ± 1.3	n.s
Gradient factor	0.67 ± 0.3	0.65 ± 0.007	n.s
Dive 2			
Maximum depth (msw)	22.1 ± 1.5	21.20 ± 0.2	n.s
Diving time (min)	50.2 ± 7.3	52.4 ± 4.4	n.s
Temperature (°C)	34.0 ± 0.9	33.7 ± 1.3	n.s
Gradient factor	0.76 ± 0.05	0.74 ± 0.01	n.s
Dive 3			
Maximum depth (msw)	40.9 ± 0.7	40.5 ± 2.9	n.s
Diving time (min)	47.8 ± 6.2	47.9 ± 2.2	n.s
Temperature (°C)	34.1 ± 0.9	33.6 ± 1.2	n.s
Gradient factor	0.82 ± 0.05	0.85± 0.05	n.s

We did not find any difference between the two groups as to anthropometric, heart rate, and diving data (Table 1).

Table 2 reports all the pre‐exposure values (before the first dive) of the COV‐19 and CTR group and the normal range value of each parameter if available. Data showed as divers start the protocol with the same values of ROS production but with a higher TAC and 8‐isoprostane levels in the CTR group. COV‐19 group showed also higher inflammation markers, with the exception of IL‐6, glycocalyx markers, creatinine and neopterin respect to CTR group.

**TABLE 2:** Comparison of basal value of biological parameters between COVID and CTR group and normal range values.

Parameter	COV‐19	CTR	*p* value	Normal value
NO related parameters				
FeNO (ppb)	16.5 ± 14.7	20.8 ± 9.9	n.s	≤25
NOx (μmol NO_3_/l)	36.9 ± 16.0	43.7 ± 25.9	n.s.	20–40
Oxidative stress				
ROS (μmol/min)	0.19 ± 0.02	0.19 ± 0.01	n.s.	n.a.
TAC (μM)	93.0 ± 30.4	120.4 ± 6.0	*	n.a.
8‐isoprostane (ng/mg creatinine)	0.20 ± 0.09	0.30 ± 0.11	*	n.a.
Albumin (g/dl)	4.4 ± 0.3	4.5 ± 0.2	n.s.	3.5–5.5
Uric Acid (mg/dl)	5.9 ± 1.1	5.6 ± 1.1	n.s.	3.5–8.5
Inflammation				
IL‐6 (pg/mL)	2.1 ± 1.2	2.2 ± 0.6	n.s.	≤5
IL‐1β (pg/mL)	7.2 ± 4.1	3.0 ± 1.0	****	≤12
TNF (pg/mL)	11.7 ± 6.4	2.7 ± 1.5	***	≤8
Glycocalyx Markers				
Heparan sulfate (μg/mL)	4.4 ± 0.9	3.3 ± 0.7	**	n.a.
Syndecan‐1 (ng/mL)	31.6 ± 9.9	15.3 ± 4.5	****	n.a.
Renal function				
Creatinine (g/dl)	0.74 ± 0.44	0.17 ± 1.17	*	0.5–1.5
Neopterin (μmol/L)	87.1 ± 59.4	58.5 ± 19.8	*	n.a.
Pulmonary parameters				
FVC (L)	5.03 ± 11	5.7 ± 0.6	n.s	4.7–5.5
FVC1 (L)	4.06 ± 0.8	4.6 ± 0.3	n.s	3.5–4.5
FEF _25/75_	3.7 ± 1.1	4.6 ± 1.2	n.s	n.a.
FEV1/FVC (%)	78.0 ± 6.9	80.7 ± 8.7	n.s	70–85
Breath rate	18.0 ± 3.4	20.1 ± 6.2	n.s	n.a
B‐Line	0.83 ± 1.3	0.46 ± 1.4	n.s	n.a

* *p* < 0.05.

** *p* < 0.01.

*** *p* < 0.001.

**** *p* < 0.0001.

### 3.1 NO derivates and oxidative stress parameters

FeNO data show no significant difference both in the COV‐19 and in the CTR group between pre‐exposure and post diving. No differences between the COV‐19 and CTR groups were found too. As to NOx, we did not find any difference between pre‐exposure values and post values both at T30 and T60 (as also reported in our previous studies (Cialoni et al., 2019, 2020)) and between the two investigated groups.

We observed a significant ROS increase at T30 of each dive in both groups. On the contrary at T60, ROS production resulted significantly increased after all dives in the COV‐19 group, but only after the third dive in the CTR group. This increase is higher in the COV‐19 group as compared to the CTR group. Furthermore, we found significant differences between the two investigated groups at both T30 and T60 as concerning the first and the second dive while the third dive showed significant differences only at T30.

We did not find any differences in the kinetics of TAC between pre‐exposure and post diving and also no differences were found between the two investigated groups.

TAC main components (albumin and uric acid) didn't show any statistically significant changes of all these components between pre‐exposure and post diving, with the exception of uric acid at T60 of the first dive. No differences were found between COV‐19 group and CTR group.

One of the most relevant findings, highlighting the between‐group differences, was the statistically significant increase in 8‐isoprostane levels at T60 in all dives of the COV‐19 group, whereas no such increase was observed in the CTR group.

No statistical difference for 8‐isoprostane between COV‐19 and CTR group was found (Table 3). Overall, NO and oxidative stress parameters comparisons were reported in Table 3.

**TABLE 3:** NO derivatives and oxidative stress markers.

Dive	COV‐19					CTR					COV‐19 vs. CTR	
	Pre	T30	*p*	T60	*p*	Pre	T30	*p*	T60	*p*	T30	T60
FeNO (ppb)												
1	16.0 ± 15.0	15.5 ± 9.0	Ns.	13.6 ± 8.5	Ns.	20.8 ± 9.9	17.7 ± 10.9	Ns.	16.9 ± 10.1	Ns.	Ns.	Ns.
2	13.4 ± 7.0	11.9 ± 6.2	Ns.	12.7 ± 8.3	Ns.	17.7 ± 10.8	16.3 ± 11.2	Ns.	17.1 ± 11.0	Ns.	Ns.	Ns.
3	13.2 ± 10.4	13.2 ± 9.6	Ns.	13.8 ± 9.6	Ns.	23.2 ± 12.5	18.0 ± 11.9	Ns.	18.1 ± 10.8	Ns.	Ns.	Ns.
NOx (μmol NO3/l)												
1	36.9 ± 16.0	42.4 ± 15.7	Ns.	39.7 ± 13.9	Ns.	43.7 ± 25.9	41.8 ± 22.8	Ns.	40.2 ± 19.3	Ns.	Ns.	Ns.
2	42.9 ± 27.7	45.8 ± 27.1	Ns.	48.8 ± 29.4	Ns.	66.0 ± 38.2	69.3 ± 41.7	Ns.	66.8 ± 41.8	Ns.	Ns.	Ns.
3	39.5 ± 14.5	39.9 ± 16.4	Ns.	39.6 ± 13.4	Ns.	40.7 ± 13.0	37.3 ± 9.6	Ns.	32.7 ± 9.6	Ns.	Ns.	Ns.
ROS (μmol/min)												
1	0.19 ± 0.02	0.30 ± 0.03	****	0.27 ± 0.03	***	0.19 ± 0.01	0.21 ± 0.01	*	0.20 ± 0.01	Ns.	****	*
2	0.21 ± 0.03	0.30 ± 0.03	****	0.28 ± 0.04	*	0.19 ± 0.01	0.24 ± 0.04	**	0.22 ± 0.03	Ns.	*	*
3	0.20 ± 0.02	0.32 ± 0.04	****	0.28 ± 0.03	***	0.20 ± 0.01	0.25 ± 0.03	**	0.23 ± 0.01	*	*	Ns.
TAC (μM Trolox eq)												
1	93.0 ± 30.4	95.7 ± 31.6	Ns.	96.6 ± 30.4	Ns.	120.4 ± 6.0	105.3 ± 9.4	Ns.	111.2 ± 8.0	Ns.	Ns.	Ns.
2	81.8 ± 41.7	88.6 ± 44.4	Ns.	76.7 ± 42.2	Ns.	115.8 ± 6.0	106.2 ± 11.7	Ns.	100.2 ± 13.2	Ns.	Ns.	Ns.
3	91.7 ± 29.9	94.7 ± 25.3	Ns.	88.2 ± 30.4	Ns.	99.8 ± 17.6	93.2 ± 8.5	Ns.	98.1 ± 10.2	Ns.	Ns.	Ns.
Albumin (g/dL)												
1	4.4 ± 0.3	4.3 ± 0.3	Ns.	4.4 ± 0.4	Ns.	4.5 ± 0.2	4.5 ± 0.4	Ns.	4.4 ± 0.2	Ns.	Ns.	Ns.
2	4.4 ± 0.4	4.4 ± 0.3	Ns.	4.3 ± 0.3	Ns.	4.4 ± 0.3	4.4 ± 0.3	Ns.	4.3 ± 0.4	Ns.	Ns.	Ns.
3	4.2 ± 0.4	4.2 ± 0.4	Ns.	4.2 ± 0.4	Ns.	4.4 ± 0.1	4.2 ± 0.5	Ns.	4.1 ± 0.4	Ns.	Ns.	Ns.
Uric Acid (mg/dL)												
1	5.9 ± 1.1	6.0 ± 1.2	Ns.	6.2 ± 1.1	Ns.	5.6 ± 1.1	5.6 ± 1.1	Ns.	6.0 ± 1.3	**	Ns.	Ns.
2	6.1 ± 1.3	6.0 ± 1.4	Ns.	6.1 ± 1.4	Ns.	5.8 ± 1.2	5.7 ± 1.2	Ns.	5.7 ± 1.2	Ns.	Ns.	Ns.
3	5.8 ± 1.0	5.7 ± 1.2	Ns.	5.9 ± 1.3	Ns.	5.8 ± 1.3	5.5 ± 1.2	Ns.	5.5 ± 1.3	Ns.	Ns.	Ns.
8‐isoprostane (ng/mg creatinine)												
1	0.20 ± 0.09	0.21 ± 0.09	Ns.	0.22 ± 0.09	**	0.30 ± 0.11	0.30 ± 0.11	Ns.	0.31 ± 0.12	Ns.	Ns.	Ns.
2	0.22 ± 0.09	0.23 ± 0.10	Ns.	0.26 ± 0.10	****	0.31 ± 0.11	0.32 ± 0.12	Ns.	0.32 ± 0.13	Ns.	Ns.	Ns.
3	0.23 ± 0.10	0.24 ± 0.09	Ns.	0.29 ± 0.09	***	0.34 ± 0.16	0.35 ± 0.16	Ns.	0.36 ± 0.16	Ns.	Ns.	Ns.

*Note*: Asterisks indicate a value significantly different.

* *p* < 0.05.

** *p* < 0.01.

*** *p* < 0.001.

**** *p* < 0.0001.

### 3.2 Inflammatory stress

Another important difference was observed for inflammatory markers (IL‐6, IL‐1β, TNF‐α) reported in Table 4. IL‐6 showed significantly increased after all dives in both T30 and T60 with the exception of the first dive in the COV‐19 group while IL‐1β exhibited significant differences at T60 after the second dive only in the COV‐19 group and at T30 and T60 after the third dive in both groups. Furthermore, TNF‐α changed significantly after all dives in the COV‐19 group with the exception of the first dive at T60 and after the second (both T30 and T60) and third dives (only T60) in the CTR group.

**TABLE 4:** Inflammatory, glycocalyx and renal function markers.

Dive	COV‐19					CTR					COV‐19 vs. CTR	
	Pre	T30	*p*	T60	*p*	Pre	T30	*p*	T60	*p*	T30	T60
IL‐6 (pg/mL)												
1	2.1 ± 1.2	3.3 ± 2.2	*	3.0 ± 1.6	Ns.	2.2 ± 0.6	2.9 ± 1.2	Ns.	2.7 ± 1.0	Ns.	Ns.	Ns.
2	2.1 ± 1.1	3.4 ± 1.7	**	3.9 ± 2.3	***	2.3 ± 0.5	2.8 ± 0.7	Ns.	2.5 ± 0.8	Ns.	Ns.	Ns.
3	2.4 ± 1.0	4.8 ± 2.2	***	6.1 ± 4.2	****	2.8 ± 0.5	4.0 ± 1.2	Ns.	3.6 ± 1.0	Ns.	Ns.	Ns.
IL‐1β (pg/mL)												
1	7.2 ± 4.1	9.1 ± 5.4	Ns.	7.4 ± 6.1	Ns.	3.0 ± 1.1	3.1 ± 1.0	Ns.	3.1 ± 1.0	Ns.	*	Ns.
2	7.4 ± 4.4	9.7 ± 5.6	Ns.	10.0 ± 5.1	**	3.0 ± 1.1	3.1 ± 1.0	Ns.	3.1 ± 1.0	Ns.	*	**
3	7.3 ± 3.9	11.1 ± 5.6	***	12.6 ± 5.3	****	3.0 ± 1.0	3.2 ± 1.0	****	3.2 ± 1.0	****	**	**
TNF‐α (pg/mL)												
1	11.7 ± 6.4	13.9 ± 7.5	Ns.	16.3 ± 10.7	***	2.7 ± 1.5	2.8 ± 1.5	Ns.	2.8 ± 1.5	Ns.	Ns.	**
2	12.3 ± 6.5	17.8 ± 7.5	*	18.6 ± 7.1	***	2.6 ± 1.5	2.9 ± 1.5	**	2.9 ± 1.5	**	***	***
3	12.0 ± 6.4	17.2 ± 7.2	***	20.8 ± 9.3	****	2.7 ± 1.5	2.8 ± 1.5	Ns.	2.8 ± 1.4	*	**	****
Heparan sulfate (μg/mL)												
1	4.4 ± 0.9	4.1 ± 0.9	Ns.	4.1 ± 0.7	Ns.	3.3 ± 0.7	3.3 ± 0.7	Ns.	3.3 ± 0.6	Ns.	Ns.	Ns.
2	4.5 ± 0.8	4.4 ± 0.8	Ns.	4.0 ± 0.8	****	3.3 ± 0.7	3.3 ± 0.7	Ns.	3.3 ± 0.6	Ns.	Ns.	Ns.
3	4.6 ± 1.0	4.4 ± 1.2	*	4.7 ± 1.4	Ns.	3.3 ± 0.7	3.3 ± 0.6	Ns.	3.3 ± 0.6	Ns.	Ns.	Ns.
Syndecan‐1 (ng/mL)												
1	31.6 ± 9.9	25.9 ± 8.8	**	26.4 ± 8.2	**	15.3 ± 4.5	15.3 ± 4.4	Ns.	15.3 ± 4.5	Ns.	*	Ns.
2	27.6 ± 8.9	23.7 ± 9.9	*	24.0 ± 7.7	*	15.3 ± 4.5	15.3 ± 4.5	Ns.	15.2 ± 4.5	*	Ns.	Ns.
3	32.2 ± 10.0	28.6 ± 10.0	Ns.	25.7 ± 10.3	***	15.3 ± 4.5	15.2 ± 4.4	Ns.	15.2 ± 4.4	Ns.	*	*
Neopterin (μmol/L)												
1	83.1 ± 57.5	106.8 ± 61.5	**	n.d	n.d.	59.5 ± 18.8	68.8 ± 18.7	Ns	n.d	n.d.	Ns.	n.d.
2	90.4 ± 60.0	135.3 ± 87.7	***	n.d	n.d.	56.6 ± 19.4	69.8 ± 21.9	*	n.d	n.d.	Ns.	n.d.
3	123.1 ± 125.7	211.04 ± 211.6	****	n.d	n.d.	64.7 ± 24.3	81.7 ± 35.5	Ns	n.d	n.d.	Ns.	n.d.
Creatinine (g/dL)												
1	0.73 ± 0.44	1.0 ± 0.5	Ns	n.d	n.d.	1.2 ± 0.5	1.5 ± 0.5	Ns	n.d	n.d.	Ns.	n.d.
2	0.74 ± 0.50	1.4 ± 0.6	***	n.d	n.d.	1.2 ± 0.5	1.7 ± 0.5	*	n.d	n.d.	Ns.	n.d.
3	0.88 ± 0.50	1.7 ± 0.6	****	n.d	n.d.	1.3 ± 0.6	1.8 ± 0.5	Ns	n.d	n.d.	Ns.	n.d.

*Note*: Asterisks indicate a value significantly different.

* *p* < 0.05.

** *p* < 0.01.

*** *p* < 0.001.

**** *p* < 0.0001.

We observed a significant higher increase of Il‐1β, TNFα in the COV‐19 group as compared with CTR group.

Heparan sulfate did not show any differences between pre‐exposure and post diving with exception at T60 in the second dive and at T60 in the third dive; no differences are found between the two investigated groups. Data about syndecan‐1 exhibited significant decreases in COV‐19 group with the exception of T30 in the first dive while CTR group exhibited significant difference only at T60 after the second dive. Finally, we observed significantly higher values in the COV‐19 group compared to the CTR group at T30 in the first and third dive and at T60 in the third dive.

As concerning renal function, neopterin exhibited significant changes after all dives in the COV‐19 group while only after the second dive in the CTR group. Creatinine changed significantly after the second and third dive in the COV‐19 group but only after the second dive in the CTR group. As concerning neopterin and creatinine, we did not observe any difference between the two investigated groups.

### 3.3 Lung function, imaging study and bubbles

As concerning lung study, we did not observe any significant difference in pulmonary function, investigated by spirometry, in breathing rate (BR) and in echographic B lines (Lung Comet) both between pre‐exposure and after the dives and between the two investigated groups as showed in Table 5.

**TABLE 5:** Lungs and heart markers investigated.

Dive	COV‐19					CTR					COV‐19 vs. CTR	
	Pre	T30	*p*	T60	*p*	Pre	T30	*p*	T60	*p*	T30	T60
FVC (L)												
1	5.0 ± 1.1	5.0 ± 1.0	Ns.	5.0 ± 1.0	Ns.	5.7 ± 0.6	5.6 ± 0.6	Ns.	5.5 ± 0.5	Ns.	Ns.	Ns.
2	5.0 ± 1.1	4.9 ± 1.0	Ns.	4.9 ± 1.0	Ns.	5.6 ± 0.6	5.8 ± 0.6	Ns.	5.6 ± 0.6	Ns.	Ns.	Ns.
3	5.0 ± 1.1	4.8 ± 0.9	Ns.	4.9 ± 1.1	Ns.	5.5 ± 0.4	5.5 ± 0.5	Ns.	5.3 ± 0.5	Ns.	Ns.	Ns.
FEV_1_ (cc)												
1	4.0 ± 0.7	3.8 ± 0.8	Ns.	3.8 ± 0.8	Ns.	4.6 ± 0.3	4.4 ± 0.4	Ns.	4.4 ± 0.4	Ns.	Ns.	Ns.
2	3.9 ± 0.8	3.9 ± 0.8	Ns.	3.9 ± 0.8	Ns.	4.5 ± 0.4	4.5 ± 0.4	Ns.	4.4 ± 0.4	Ns.	Ns.	Ns.
3	3.9 ± 0.8	3.7 ± 0.7	Ns.	3.8 ± 0.8	Ns.	4.5 ± 0.3	4.4 ± 0.4	Ns.	4.3 ± 0.4	Ns.	Ns.	Ns.
FEF_25‐75_ (L/s)												
1	3.5 ± 1.0	3.5 ± 0.8	Ns.	3.5 ± 1.0	Ns.	4.6 ± 1.2	4.2 ± 1.1	Ns.	4.5 ± 1.1	Ns.	Ns.	Ns.
2	3.5 ± 0.9	3.6 ± 0.9	Ns.	3.6 ± 0.8	Ns.	4.6 ± 1.1	4.2 ± 1.3	Ns.	4.6 ± 1.3	Ns.	Ns.	Ns.
3	3.7 ± 0.9	3.4 ± 0.9	Ns.	3.5 ± 0.9	Ns.	4.7 ± 1.4	4.7 ± 1.4	Ns.	4.6 ± 1.3	Ns.	Ns.	Ns.
FEV_1_/FVC (%)												
1	77.5 ± 6.7	76.6 ± 6.8	Ns.	76.0 ± 8.3	Ns.	80.7 ± 8.7	79.4 ± 9.0	Ns.	80.8 ± 9.0	Ns.	Ns.	Ns.
2	77.9 ± 5.0	78.3 ± 4.0	Ns.	78.3 ± 3.9	Ns.	81.0 ± 9.2	78.1 ± 10.6	Ns.	80.4 ± 8.8	Ns.	Ns.	Ns.
3	78.9 ± 4.0	77.6 ± 5.8	Ns.	77.7 ± 5.3	Ns.	80.8 ± 8.8	81.7 ± 9.1	Ns.	81.8 ± 9.3	Ns.	Ns.	Ns.
Breath rate (bmp)												
1	18 ± 3.5	18.4 ± 2.3	Ns.	n.d	n.d.	20.1 ± 6.2	20.1 ± 4.9	Ns.	n.d	n.d.	Ns.	n.d
2	20.9 ± 1.9	19.1 ± 2.0	Ns.	n.d	n.d.	21.3 ± 5.5	20.4 ± 5.4	Ns.	n.d	n.d.	Ns.	n.d
3	20.0 ± 2.9	19.3 ± 3.2	Ns.	n.d	n.d.	21.6 ± 5.1	20.0 ± 3.6	Ns.	n.d	n.d.	Ns.	n.d
Lung Comet (*n*)												
1	0.8 ± 1.3	1.7 ± 2.3	Ns.	0.4 ± 1.2	Ns.	0.8 ± 1.4	0.4 ± 0.9	Ns.	0.2 ± 0.7	Ns.	Ns.	Ns.
2	1.0 ± 1.7	1.6 ± 2.4	Ns.	1.5 ± 2.4	Ns.	0.0 ± 0.0	0.0 ± 0.0	Ns.	0.0 ± 0.0	Ns.	Ns.	Ns.
3	1.0 ± 1.5	1.3 ± 2.1	Ns.	0.1 ± 0.4	Ns.	0.0 ± 0.0	0.3 ± 0.7	Ns.	0.0 ± 0.0	Ns.	Ns.	Ns.
Bubble (*n*)												
1	0.0 ± 0.0	0.1 ± 0.4	Ns.	0.0 ± 0.0	Ns.	0.0 ± 0.0	0.0 ± 0.0	Ns.	0.0 ± 0.0	Ns.	Ns.	Ns.
2	0.0 ± 0.0	0.5 ± 1.1	Ns.	0.3 ± 0.9	Ns.	0.0 ± 0.0	1.0 ± 1.0	Ns.	0.4 ± 0.5	Ns.	Ns.	Ns.
3	0.0 ± 0.0	0.7 ± 1.0	Ns.	0.5 ± 0.9	Ns.	0.1 ± 0.3	1.1 ± 1.5	Ns.	1.6 ± 1.3	Ns.	Ns.	Ns.

Additional, we did not find any differences in bubble formation between the two investigated groups.

No significant variations in ECG, respiratory rate, posture, or arterial oxygen saturation were observed across the time points defined by the experimental protocol, as recorded by the wearable system.

## 4 DISCUSSION

In the present study, individuals with a past history of COVID‐19 were employed as an experimental model of pulmonary infection to identify biomarkers of persistent structural and functional abnormalities, both systemic and lung‐specific, that may remain beyond clinical recovery. Furthermore, we investigated the effects on the return to scuba activity. To expose the subjects, previously affected by pulmonary infection, we used SCUBA diving as a model of dysbaric stress because it is very easy to do, as compared to high altitude or space, and because the physiological adaptations related to diving are well known and repeatable. Subjects enrolled in the protocol were all recently affected by COVID‐19 syndrome with similar moderate symptoms (fever, cough, chest pain), but none of them needed intensive care.

The differences in initial antioxidant defense levels (higher in the CTR group), as well as inflammatory and glycocalyx markers (higher in the COV‐19 group) suggest the effects of COVD‐19 symptoms may not be fully resolved even 3 months after recovery.

We did not find any difference in FeNO levels before and after any dive and between the groups; this could be related to the flow rate of our devices (50 mL/s) has determined a major measurement of the conductive way and only in a low percentage of the alveolus air (Cameli et al., 2021). Also, we did not observe substantial differences in the management of diving related vascular work in the subjects, as shown by the similar NOx levels found in the two groups after diving. As observed previously after dives at 40 m (Cialoni et al., 2019, 2020), NOx levels normally return to pre‐diving values to restore the NO consumed during the dive within 60 min through various metabolic pathways, including xanthine oxidase and xanthine oxidoreductase (Godber et al., 2000; Jansson et al., 2008; Li et al., 2003). NO plays a key role in regulating blood vessel diameter and flow, acting also as a signaling molecule for regulating vascular tone (Jin & Loscalzo, 2010).

However, the absence of significant differences in FeNO and NOx between groups should be interpreted with caution, although these findings remain suggestive and physiologically interesting. From a physiological perspective, NO bioavailability reflects the balance between its synthesis and inactivation, and increased oxidative stress may rapidly scavenge NO. Consequently, apparently normal NOx levels could mask compensatory mechanisms of NO overproduction. Moreover, single‐flow FeNO measurements predominantly reflect NO derived from the proximal airways, potentially underestimating alveolar or endothelial NO dynamics.

Oxidative stress is promoted by the hyperbaric‐related high pO_2_, which increases free O_2_, potentially favoring mitochondrial uncoupling and serving as an independent source of ROS/RNS (Ottolenghi et al., 2020). We observed higher ROS levels in both groups after diving, with a statistically significant greater increase in the COV‐19 group, likely reflecting residual immune activation from prior infection (Zhang et al., 2017). ROS interact with biological macromolecules, triggering oxidative stress and activating NF‐κB and Nrf2 pathways (Fratantonio et al., 2021; Mrakic‐Sposta et al., 2020). SCUBA divers consequently activate endogenous antioxidant defenses to limit oxidative stress and prevent excessive depletion (Brizzolari et al., 2025; Cialoni et al., 2019; Mrakic‐Sposta et al., 2024; Sureda et al., 2012), providing adaptive protection through ROS scavenging and maintenance of NO production, avoiding eNOS inhibition (Kuzkaya et al., 2003; Rothfuss et al., 1998). Basal TAC values were higher in the CTR group, probably because the lack of exposure to SARS‐CoV‐2 maintained more efficient mechanisms of antioxidant defenses. On the other hand, we found a similar antioxidant response in the two groups, confirmed also by the analysis of TAC components, albumin and uric acid. Anyway, the antioxidant response involves very complex mechanisms, not permitting us to clarify further details with a single study. Despite TAC levels, we observed lower levels of 8‐iso‐PGF2α, a well‐known marker of oxidative stress damage (Morrow et al., 1990), in the COV‐19 group than in the CTR group, confirming the antioxidant response. This may be related to oxidative stress role in normal cell physiology by regulating redox‐sensitive signaling NF‐κB and Nrf2/Keap1 pathways (D'Autreaux & Toledano, 2007; Itoh et al., 1997; Schreck et al., 1991). This aspect seems to confirm a major effect of oxidative stress in COVID‐19 subjects that agrees with the lower antioxidant response found.

Oxidative stress results in the release of inflammatory markers including IL‐1β, IL‐6, and TNF‐α (Bosco et al., 2018), leading to the activation of inflammation cascades (Bigley et al., 2008; Hennigar et al., 2017; Zarak et al., 2021). Indeed, IL‐6, IL‐1β, and TNF‐α levels are elevated in the COVID‐19 acute inflammation phase (Koutsakos et al., 2021), suggesting a role as mediators of an ongoing SARS‐CoV‐2‐directed immune response (Schultheiss et al., 2022). Therefore, the increase of inflammation markers in the COV‐19 group could be very important to manage the return to extreme exposure in subjects affected by COVID‐19. Indeed, this elevated value might reflect a persistent low‐grade inflammatory state that could exacerbate oxidative stress responses during hyperbaric exposure. Such a condition might increase susceptibility to endothelial dysfunction (Gao et al., 2022), impair vascular reactivity (Jud et al., 2021), and may promote a cardio‐respiratory strain when challenged by elevated pO_2_ levels.

As consequence of inflammation status, glycocalyx indexes, heparan sulfate and syndecan‐1 decreased in COV‐19 group due to their degradation by ROS/RNS and pro inflammatory markers. Heparan sulfate plays a key role in the interaction with the spike protein of SARS‐CoV‐2, facilitating the initial binding of the virus to host cells (Zhang et al., 2024). In our subjects, we did not observe notable changes, probably due to mild disease entity. We observed a reduction of Syndecan‐1 value in group after each dive. Loss of Syndecan‐1 may be related to the onset of inflammatory status (Voyvodic et al., 2014), resulting from endothelial function alteration. Indeed, COV‐19 group exhibited higher values of pro inflammatory markers respect to CTR group.

Neopterin concentration can rise during systemic oxidative stress (Brizzolari et al., 2023; Mrakic‐Sposta et al., 2019) with more pronounced changes in COV‐19 group where we observed a greater ROS and inflammation markers production.

Data on changes in glycocalyx‐related markers provide additional insight into the potential endothelial alterations observed in post‐COVID subjects. The endothelial glycocalyx plays a crucial role in vascular homeostasis, including the regulation of shear stress signaling, vascular permeability, and leukocyte–endothelium interactions. Because oxidative stress and inflammatory mediators can promote glycocalyx degradation, these alterations may occur even before overt vascular dysfunction becomes detectable. In the context of diving, where hyperoxia and increased shear stress challenge endothelial stability, subtle glycocalyx impairment may contribute to increased susceptibility to microvascular stress and inflammatory responses.

About pulmonary response, the investigated spirometry parameters about pulmonary function did not show differences between the two groups, suggesting COVID‐19 did not leave lung damage and/or reduced capacity in our subjects. Furthermore, COV‐19 subjects tend to develop a higher number of pulmonary comets compared to the CTR group and this might be related to a residual inflammation status and/or to a more pronounced susceptibility to lung inflammation. In post‐COVID divers, hyperbaric exposure and residual post‐infectious alterations appear to act additively, amplifying oxidative stress during and after immersion. The combination of hyperoxia, lingering immune activation, and temporarily reduced antioxidant capacity may enhance ROS production, activate redox‐sensitive signaling pathways, and promote cytokine release. Importantly, this represents a functional susceptibility that could not be easily detectable by standard baseline pulmonary tests and could therefore fail to fully capture physiological risk under hyperbaric stress. Among the investigated groups, no significant variations in ECG, respiratory rate, and arterial oxygen saturation recorded by the wearable system were observed, suggesting that, despite the environmental and physical challenges, basic physiological parameters remained stable.

Overall, our data suggest that repetitive dives in the COV‐19 group lead to a greater release of ROS/RNS, likely triggered by increased pO_2_, which in turn results in an elevated inflammatory response. Given the similarities in physiological responses across various extreme environments, it is likely that these findings could be confirmed under other environmental conditions as well. Furthermore, we observed a major inflammatory response in the COV‐19 group after diving as compared to the CTR group, indicating a different response in the two groups even if COV‐19 respects the guideline of 3 months of time before resuming activities.

As concerning the return to SCUBA diving after COVID‐19 we have to consider the differences in inflammatory responses observed between the two investigated groups. These observed differences underscore the importance of exercising caution and conducting personalized assessments, particularly with regard to the severity of the COVID‐19 illness and the individual's recovery trajectory. In our study, we were also able to evaluate directly the cardiopulmonary and oxidative stress responses to SCUBA diving in subjects who had previously contracted COVID‐19 and who met existing return‐to‐diving guidelines. Until now, only broad consensus statements have been issued by expert groups such as the European Underwater and Baromedical Society (EUBS) (European Underwater and Baromedical Society, 2020), the Undersea and Hyperbaric Medical Society (UHMS) (Undersea & Hyperbaric Medical Society, 2020) and Divers Alert Network (DAN) Europe (Divers Alert Network (DAN) Europe, 2020). Although our results align with prior recommendations suggesting a minimum three‐month recovery interval before resuming SCUBA diving post‐COVID‐19 pulmonary infection (Mirasoglu et al., 2022; Morin et al., 2022) we caution that this timeframe should not be interpreted as universally applicable. Indeed, the potential for prolonged subjective symptoms and variable physiologic recovery trajectories argues in favor of individualized medical evaluations conducted by hyperbaric medicine physicians, followed by a carefully structured dive plan with incremental increases in depth. Accordingly, we strongly advocate for a formal fitness‐to‐dive (FTD) assessment, even in asymptomatic individuals recovering from COVID‐19, preferably involving a collaborative follow‐up regimen integrating both pulmonary medicine and diving medicine specialists. According to Morin et al., 3 months after symptoms disappear with FTD positive evaluation may be a reasonable period without any lung and cardiac anomaly (Morin et al., 2022).

As regards the tests carried out in the protocol, we must highlight a limitation in the use of a single exhalation flow rate to measure FeNO; this aspect will need an in‐depth analysis in future studies probably using a multiple flow technique (Hemmingsson et al., 2004; van Ooij et al., 2010). Furthermore, we should also consider a limitation: all the divers were affected by the Alpha variant of COVID‐19, so the findings cannot be generalized to other variants. Our findings of elevated IL‐1β and TNF‐α in post‐COVID‐19 divers could reflect a post‐infectious, low‐grade inflammatory phenotype rather than overt pulmonary disease. Considering that hyperbaric exposure increases vascular shear stress and oxygen partial pressure, and even subtle endothelial inflammation may impair microvascular regulation, potentially altering vascular reactivity and oxidative susceptibility without detectable changes in the spirometry. Accordingly, these results underscore that FTD evaluations cannot rely solely on pulmonary function testing, but should include a broader assessment of cardiovascular and inflammatory status in post‐infectious individuals.

Finally, another limitation is the small sample size that does not permit us to consider our finding representative of the diving population.

As concerning strength points, this study provides valuable insights into the physiological and inflammatory responses of subjects recovering from COVID‐19 when exposed to SCUBA diving focusing on oxidative stress, vascular function, and inflammatory markers, offering a robust framework for understanding post‐COVID‐19 recovery under such conditions, trying to build guidelines for returning to extreme environment activities.

## 5 CONCLUSION

The present investigation demonstrates that individuals with a history of COVID‐19, even after apparent clinical recovery and adherence to the current three‐month return‐to‐diving guideline, may still exhibit heightened oxidative and inflammatory responses when exposed to hyperbaric stress. It should be noted that all participants were infected with the SARS‐CoV‐2 Alpha variant and unvaccinated, which may limit generalizability to later variants and vaccinated populations, even if it can offer a general indication to the return to diving after pulmonary infection.

Although no persistent alterations in spirometric parameters or overt impairment of pulmonary function were identified, the evidence of increased ROS production, augmented inflammatory marker release, and changes in glycocalyx integrity strongly suggests the persistence of subtle systemic and vascular alterations. These findings emphasize the need for caution and underline the importance of a personalized, multidisciplinary approach to FTD assessments, integrating expertise in pulmonary and diving medicine. Further and larger studies, including subjects affected by different SARS‐CoV‐2 variants, will be essential to confirm and extend these observations. Ultimately, our data contribute to the growing body of evidence supporting the development of specific guidelines for the safe return to SCUBA diving and other extreme environmental exposures in individuals recovering from COVID‐19.

## AUTHOR CONTRIBUTIONS

**Danilo Cialoni:** Conceptualization; data curation; investigation; methodology; project administration; supervision; validation; visualization. **Andrea Brizzolari:** Data curation; formal analysis; investigation; methodology. **Simona Mrakic‐Sposta:** Data curation; formal analysis; investigation; methodology; validation. **Alessandra Vezzoli:** Data curation; formal analysis. **Cinzia Dellanoce:** Formal analysis. **Massimo Pieri:** Investigation. **Riccardo Pelliccia:** Investigation. **Chiara Petrassi:** Investigation. **Gerardo Bosco:** Validation; visualization. **Alessandra Barassi:** Formal analysis. **Alessandro Marroni:** Conceptualization; project administration; resources; supervision; visualization.

## FUNDING INFORMATION

This study was funded by DAN Europe Foundation, funded internally by the Hyperbaric Medicine School of the Department of Biomedical Sciences at the University of Padova and the Institute of Clinical Physiology (Milan) National Research Council (IFC‐CNR).

## CONFLICT OF INTEREST STATEMENT

The authors declare no conflict of interest in completing this research study.

## ETHICS STATEMENT

This study was conducted in accordance with the Helsinki Declaration and was approved by the Ethycal Commitee of Università degli Studi di Milano, Italy (Aut. N°37/17).

## CONSENT STATEMENT

Written informed consent was obtained from all subjects involved in the study.

## Data Availability

Dataset used in this study can be made available on a reasonable request basis. Requests must include an appropriate protocol, analysis plan, and data exchange with institutional approvals in place prior to data transfer of any information.
